# Hybrid Assembly from a Pathogenic Methicillin- and Multidrug-Resistant Staphylococcus pseudintermedius Strain Isolated from a Case of Canine Otitis in Spain

**DOI:** 10.1128/MRA.01121-19

**Published:** 2020-01-02

**Authors:** Joaquim Viñes, Anna Cuscó, Olga Francino

**Affiliations:** a Servei Veterinari de Genètica Molecular (SVGM), Universitat Autònoma de Barcelona, Bellaterra, Cerdanyola del Vallès, Barcelona, Spain; b Vetgenomics SL, Bellaterra, Cerdanyola del Vallès, Barcelona, Spain; Indiana University, Bloomington

## Abstract

Here we report the genome assembly, using a hybrid approach with Illumina and Nanopore sequencing, of a pathogenic Staphylococcus pseudintermedius strain isolated from a case of canine otitis. Genome assembly confirmed the antimicrobial resistance profile (disk diffusion testing) with specific genes and mutations.

## ANNOUNCEMENT

Staphylococcus pseudintermedius is a coagulase-positive *Staphylococcus* species within the Staphylococcus intermedius group (1), which is formed by S. intermedius, S. pseudintermedius, S. delphini, and S. cornubiensis (2). This bacterium is a commensal in pets’ microbiota, typically related to dogs and primarily associated with skin, fur, and mucocutaneous sites. However, it has opportunistic behavior, causing several types of infections related mostly to the skin, such as wound infections, pyoderma, and otitis, among others. S. pseudintermedius colonizes 90% of dogs, and methicillin-resistant S. pseudintermedius (MRSP) demonstrates high prevalence worldwide, e.g., 70% in Japan, 50% in China, and 30% in Europe. The report of zoonotic infections due to MRSP highlights its One Health threat. Sequence type 71 (ST71) is the most prevalent in Europe (3–8).

Swab extension of the sample and further Diff-Quick staining revealed the presence of a few cocci in the left ear of a 6-year-old Yorkshire terrier with otitis externa. The S. pseudintermedius G3C4 strain was isolated by overnight culture at 37°C on blood agar. Different antibiotics were tested in a disk diffusion inhibitory assay, with the sensitivity range criteria shown at Table 1 for aminoglycosides, fluoroquinolones, tetracyclines, macrolides, beta-lactams, lincosamides, phenicols, rifamycins, fusidic acid, and co-trimoxazole.

**TABLE 1:** Summary of the antibiotic resistance determined by disk diffusion testing and sequencing

Antibiotic^*a*^	Disk diffusion testing results			Sequencing results		
	Zone of inhibition (mm)	Sensitivity range (μg/ml)	Susceptibility^*b*^	Gene(s) associated	Mutation associated	Location
Aminoglycosides						
GEN	12	<12 to >15	R	*aac(6′)-Ie-aph(2ʺ)-Ia*, *aph(3′)-IIIa*, *aad(6)*		Genome
TOB	17	<17 to >19	R			
AMK	25	<14 to >17	S			
Fluoroquinolones						
CIP	9	<20 to >22	R		Point mutations in *gyrA* (positions 12, 251, 2023, and 2140)	Genome
MARBO	0	<14 to >20	R			
PRADO	15	<19 to >24	R			
ORBI	0	<17 to >23	R			
Tetracyclines						
TET	10	<18 to >23	R	*tet*(K)		Plasmid
DOX	14	<20 to >25	R			
MIN	18	<19 to >24	R			
Macrolide						
ERY	0	<13 to >23	R	*ermB*		Genome
Beta-lactams						
OXA	0	<16 to >18	R	*mecA*, *blaZ*		Genome
FOX	30	<34 to >36	R			
Lincosamide						
CLI	0	<14 to >21	R	*ermB*		Genome
Phenicols						
CHL	34	<12 to >18	S			
FFC	30	<12 to >18	S			
Rifamycin						
RIF	44	<16 to >20	S			
Fusidane						
FD	40	<23 to >25	S			
SXT	0	<10 to >16	R	*dfrG*		Genome

a GEN, gentamicin; TOB, tobramycin; AMK, amikacin; CIP, ciprofloxacin; MARBO, marbofloxacin; PRADO, pradofloxacin; ORBI, orbifloxacin; TET, tetracycline; DOX, doxycycline; MIN, minocycline; ERY, erythromycin; OXA, oxacillin; FOX, cefoxitin; CLI, clindamycin; CHL, chloramphenicol; FFC, florfenicol; RIF, rifampin; FD, fusidic acid; SXT, co-trimoxazole.

b R, resistant; S, sensitive.

DNA from S. pseudintermedius G3C4 was extracted using a DNA microprep kit (ZymoBIOMICS), following the manufacturer’s instructions, and DNA quality was assessed by measurements with a Qubit fluorimeter (Invitrogen). The library for Nanopore sequencing was prepared by transposase fragmentation using a rapid barcoding kit (product number RBK-SQK004; Oxford Nanopore Technologies), according to the manufacturer’s instructions. The final library was loaded and sequenced in a MinION FLO-MIN106 flow cell v9.4.

An Illumina (San Diego, CA, USA) library was prepared by enzymatic fragmentation and double indexing using an NGSgo kit (GenDx, Utrecht, Netherlands), according to the manufacturer’s instructions. The indexed libraries were pooled, denatured, and diluted to a final concentration of 4 nM. The pooled library was sequenced on the MiSeq system (Illumina) with a 300-cycle MiSeq reagent kit v2.

The fast5 files generated by Nanopore sequencing were base called and demultiplexed (sorted by barcode) using Albacore v2.3.1, yielding fastq files. A second round of demultiplexing was performed with Porechop (9) (by default), in which barcodes that agreed with Albacore were kept and the others were removed. Porechop was also used to trim barcodes and other adapters from the sequences. A total of 93,340 Nanopore reads were retrieved and used for further steps; the median read length was 2,774 bp, the *N*_50_ read length was 4,382 bp, and the median Phred read quality was 14.2. A total of 2,338,855 Illumina reads were generated by the sequencer, with a median Phred read quality of 37.8. *De novo* genome assembly was performed with data retrieved from Nanopore and Illumina sequencing in a hybrid approach, using Unicycler v0.4.6 (10) (parameters were as follows: R1, Illumina file; R2, Illumina file; l, Nanopore reads). Further analyses included assessment of genome completeness with CheckM v1.0.11 (11) (by default), multilocus sequence typing (MLST) using CGE DTU tools (12), and annotation with Prokka v1.13 (13) (by default); the NCBI Prokaryotic Genome Annotation Pipeline v4.6 was used to determine the number of coding sequences, rRNAs, and tRNAs. We used ABRicate (14) with the CARD and NCBI databases to retrieve antibiotic resistance genes.

Unicycler assembly retrieved 7 contigs. Two of the contigs corresponded to the complete genome and a plasmid, with 60× coverage and lengths of 2.72 Mb and 4.4 kb, respectively. The 5 other contigs had coverage between 1.71× and 1.83×.

The assembled genome of this S. pseudintermedius isolate (63× coverage) has a size of 2,717,621 bp, with a G+C content of 37.50% and 2,548 coding sequences, 59 tRNAs, and 19 rRNA copies. CheckM determined completeness of 99.43%. MLST (12) showed that the strain belongs to the most prevalent ST in Europe, ST71, achieving 100% coverage and identity for all of the genes tested (*ack*, *cpn60*, *fdh*, *pta*, *purA*, *sar*, and *tuf*).

A plasmid of 4,439 bp, pSP-G3C4, was also obtained. It has a G+C content of 30.07% and 64× coverage. In a BLAST search, we obtained a match with Staphylococcus epidermidis ATCC 12228 plasmid pSE-12228-01 (NCBI accession number NC_005008).

Table 1 shows the results of disk diffusion susceptibility tests with aminoglycosides, fluoroquinolones, tetracyclines, macrolides, beta-lactams, clindamycin, and co-trimoxazole. Genome analyses with ABRicate revealed the presence of several genes that confer resistance to most of the aforementioned antibiotics, including *blaZ* and *mecA* for beta-lactam resistance, *aac(6′)-Ie-aph(2ʺ)-Ia*, *aph(3′)-IIIa*, and *aad(6)* for aminoglycoside resistance, *ermB* for erythromycin and clindamycin resistance, and *dfrG* for trimethoprim resistance. We also found *sat4*, which confers resistance to streptothricin. Point mutations at positions 12, 251, 2032, and 2140 in the *gyrA* gene (encoding a topoisomerase) explain quinolone resistance (15). The *tet*(K) gene, conferring resistance to tetracycline, was found in the plasmid.

A SCC*mec* II-III cassette characteristic of S. pseudintermedius (15) harbors the methicillin resistance gene *mecA*. Furthermore, *aad(6)*, *sat4*, and *aph(3′)-IIIa* genes are located contiguously in the genome, which is an antibiotic resistance gene cluster already described for this species (16). It seems that a fourth gene could be involved in the cluster, namely, *ermB*, which is located near the triad of genes mentioned previously. Boerlin et al. (17) already reported that there could be a link between macrolide and aminoglycoside resistance in *Staphylococcus* strains of canine origin.

We confirm that a long- and short-read hybrid approach is an excellent option for sequencing and assembling *de novo* genomes for in-depth assembly and characterization.

### Data availability.

The genome sequence of S. pseudintermedius G3C4 has been deposited in the GenBank database with accession number CP032682 and RefSeq accession number NZ_CP032682; the plasmid has been deposited under GenBank accession number MN612109. All raw sequence files can be found under BioProject accession number PRJNA493792.
